# Ultrasonic Thermal-Assisted Extraction of Phosvitin from Egg Yolk and Evaluation of Its Properties

**DOI:** 10.3390/polym11081353

**Published:** 2019-08-15

**Authors:** Bin Jiang, Linlin Wang, Xiaojing Wang, Shuang Wu, Dongmei Li, Chunhong Liu, Zhibiao Feng

**Affiliations:** 1 Department of Applied Chemistry, Northeast Agricultural University, NO.600 Changjiang Road Xiangfang District, Harbin 150030, China; 2 Heilongjiang Eco-Meteorology Center, NO.71Diantan Road Xiangfang District, Harbin 150030, China

**Keywords:** ultrasonic-thermal assisted extraction, Phosvitin, extraction, evaluation

## Abstract

Phosvitin (Pv) is the principal phosphoprotein in chicken egg yolk and the most highly phosphorylated protein in nature. Pv is a good natural food antioxidant and emulsifier. However, the current extraction methods present disadvantages of complicated operation and are time-consuming. In this paper, Pv was extracted from the egg yolk by ultrasonic thermal-assisted extraction (UTAE). The effects of heating time, ultrasonic power and ultrasonic time on the extraction of Pv were investigated by a single factor. The purity of Pv, ratio of nitrogen to phosphorus (N/P), and activity were used as evaluation indexes. An efficient extraction of Pv was achieved when the sample was heated for 15 min at 80 °C and then processed for 10 min of ultrasonic treatment with an ultrasonic power of 600 W. Under optimal conditions, the purity and activity of Pv were 80% and 98%, respectively, whereas the ratio of N/P was 3.1. The obtained Pv was identified by sodium dodecyl sulfate–polyacrylamide gel electrophoresis (SDS-PAGE), Fluorescence analyses, fourier-transform infrared (FT-IR), and liquid chromatography-nanoelectrospray ionization mass spectrometry (Nano LC-ESI-MS/MS) analysis. The results showed there is no significant difference in the properties of Pv obtained by UTAE and Pv standard. The developed extraction approach is a simple, industrial compatible method without the use of any organic solvents.

## 1. Introduction

Phosvitin (Pv) is derived from the cleavage product of vitellogenin (VIT) and has many properties which are used as functional food ingredients [1]. Pv accounts for about 11% of egg yolk protein and 4% of the dry weight of egg yolk; Pv is the major phosphoprotein in egg yolk [2] and a highly phosphorylated protein [3]. Pv contains 217 amino acid residues, of which 123 are serine [4] and more than 80% of serine is phosphorylated. Pv has three hydrophobic residues at the C-terminus and nine hydrophobic residues at the N-terminus, thus forming an amphiphilic structure with intermediate hydrophilic and hydrophobic ends. Pv’s unique phosphorylation structure has many properties. Pv is reported to exhibit various physiological activities such as heat stability [5,6], binding of metal [7,8,9], antioxidant activity [10,11,12], and emulsifying properties [13,14,15], indicating its promising application as functional food ingredient or nutraceutical. Egg yolk proteins are commonly used as food ingredients because of their excellent emulsification. Being a highly phosphorylated protein present in the egg yolk, Pv has an affinity for lipids to form an excellent emulsion during the emulsification [15]. In 1949, Pv was first isolated from egg yolk by Mecham et al. [5] using ether organic solvent separation with a recovery rate ranging from 60% to 70%. At present, most methods for separating Pv use non-food-grade solvents, such as chloroform, methanol and ether, to remove lipids in granules from egg yolk, and thus the obtained Pv cannot be used by humans. In addition, the structure of Pv is denatured due to the use of solvents, which results in low Pv recovery and loss of native functions [16]. In recent years, several methods have been published for separating Pv without using toxic solvents. In 2011, Lei et al. [17] used QFF anion exchange chromatography to separate and purify Pv in egg with a purity of 92.6% and a yield of total Pv in yolk of 35.4%. Polyethylene glycol and anion exchange chromatography was used to separate Pv after a two-step salt precipitation, and the purity of the obtained Pv reached 99% [18]. Although the purity of Pv obtained using an ion exchange chromatography was satisfactory, the operation was complicated and the yield was low. In 2015, Pv was extracted by heat treatment with 10% NaCl at 90 °C by Ren et al. [19]. Under the optimum conditions, the purity of Pv was 88.0% and the recovery was 58.8%. Although it was of high purity, it had low recovery and was not suitable for mass production. In 2015, Zhang et al. [18] developed a new preparation method for Pv, which obtained high purity Pv (99%) with a recovery of 47% under the optimal condition of pH 4.0 and 3% PEG 6000. It was a mild method with high purity, but the complicated process limited further application. At present, most of the methods have suffered disadvantages such as residual organic solvents, complicated operation, time-consuming, and difficult to expend. It is necessary to explore a new extraction method.

At present, new methods that could improve the extraction efficiency of proteins, such as enzymatic [20], ultrasound [21], microwave [22], pulsed electric field [23], and supercritical fluid extraction [24], have been performed by researchers [25]. Ultrasound-assisted extraction was widely used in the extraction of bioactive substances due to several advantages including time saving, high crushing efficiency, high extraction efficiency, no organic reagent residue, and energy saving [26]. In ultrasound-assisted extraction, the ultrasound accelerated mass transfer during the process, causing proteins to be released from the parenchyma cell wall by cavitation [27]. The high extraction efficiency of ultrasound-assisted extraction was mainly due to its mechanical effect. The mass transfer between immiscible phases was greatly facilitated by super agitation [28]. Micro-jetting and micro-streaming was regarded as the most important mechanical effects of ultrasonic treatments [29,30]. Vernes et al. [31] reported the application of ultrasound-assisted in the extraction of proteins from dry *Arthrospira platensis* cyanobacteria. Compared to the conventional process without ultrasound, the recovery rate (28.42 ± 1.15 g/100 gDW) was 2.3 times that of the conventional process (8.63 ± 1.15 g/100 gDW), and the ultrasound-assisted extraction effect was obviously better than the traditional extraction method. Tomas et al. [32] used different methods to extract proteins from *Ganxet* beans (*Phaseolus vulgaris* L. var. *Ganxet*). In general, ultrasound treatment resulted in higher yields and increased percentages of dissolved substances and protein recovery. To effectively increase protein recovery, Ahmet et al. [25] combined enzymatic and ultrasound-assisted extraction methods to separate protein and antioxidant compounds from sesame bran. Ultrasound was considered to be an effective means to improve the extraction efficiency. Ultrasound was economically feasible and meets the process requirements including amplification. Therefore, the extraction method adopted in this study is ultrasonic thermal-assisted extraction (UTAE).

Although the use of an ultrasound has been well documented in the literature [31,32], few applications of ultrasounds in extracting proteins from egg yolk have been reported. The aim of the current work was to develop an UTAE technique for separating Pv from egg yolk without using toxic solvents. Also, the developed method was aimed at large-scale production and to be appropriate for human use with high yield and purity.

## 2. Materials and Methods

### 2.1. Reagents

The egg sample was bought from local supermarkets. Pv standard from egg yolk was obtained from Sigma Chemical Company (St. Louis, MO, USA). Sodium dodecyl sulfate–polyacrylamide gel electrophoresis (SDS-PAGE) gel preparation kit (Solarbio, Beijing, China) was used for characterization of the protein. All other chemicals obtained from Aladdin (Shanghai, China) were of analytical grade. All the solutions were prepared using ultrapure water obtained from Northeast Agricultural University.

### 2.2. Determination of the Process Parameters for Pv Isolation

**Figure 1:**
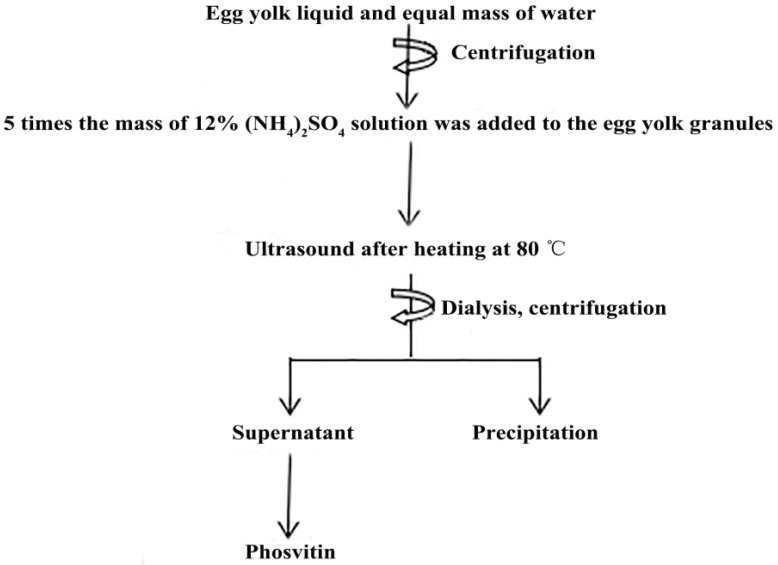
Isolation procedure of Pv.

The process of preparing Pv is shown in Figure 1. The egg yolk was separated from the egg white using an egg yolk separator and rolled on the filter paper slowly. Then a needle was used to pierce the egg yolk membrane, and the shed egg yolk solution was collected into the beaker. The egg yolk solution was mixed with an equal mass of ultrapure water and stirred for 1 h in an ice water bath. The obtained egg yolk solution was centrifuged at 10,000× *g* for 45 min, and the precipitate was collected to obtain egg yolk granules. The obtained egg yolk particles were weighed and then (NH_4_)_2_SO_4_ solution with five times the weight of egg yolk was added and stirred in an ice water bath for 3 h. According to the preliminary experiment and literatures, 80 °C was chosen for treating the mixture. After being stored at 4 °C over night, the mixture was stirred and heated in a water bath at 80 °C for 0 min, 5 min, 10 min, 15 min, and 20 min respectively to study the effect of heating time on the extraction efficiency. After being treated by ultrasound with ultrasound power of 600 W for 10 min—the Ultrasonic Homogenizer was from Ningbo Xinzhi Biotechnology Co., Ltd. (Ningbo, China)—the mixture was dialyzed against running water for 12 h. Centrifugation was performed at 10,000× *g* for 25 min, and the supernatant was collected and lyophilized (LyoQuest-85 Plus, Telstar, Spain) to obtain Pv lyophilized powder which was used to prepare Pv sample solution or for further characterization. An ultrasound time of 0 min, 5 min, 10 min, and 15 min and ultrasound power of 400 W, 500 W, 600 W, and 700 W were performed to study the effect of ultrasound time and ultrasound power on the extraction efficiency, respectively. The extraction efficiency was evaluated with purity, ratio of nitrogen to phosphorus (N/P), and activity. Pv is a protein with high phosphorus content, and the N/P value could reflect the purity of Pv to some extent [5].

### 2.3. Determination of Purity

The total nitrogen and inorganic nitrogen in the extracted Pv sample were determined by Kjeldahl method with automatic Kjeldahl analyzers (K9840, Hanon Instrument, Jinan, China) [33]. Samples of inorganic nitrogen were undigested samples.

Nitrogen content in samples was calculated with the following formula:

$$\begin{array}{c} N_{1}=\frac{\left(V_{1}-V_{2}\right)\times C\times 0.014}{V}\\ N_{2}=\frac{\left(V_{1}-V_{2}\right)\times C\times 0.014}{V}\\ N=N_{1}-N_{2} \end{array}$$

where *N*_1_ was the total nitrogen content in the sample; *N*_2_ was the content of inorganic nitrogen in the sample; *N* was the nitrogen content in the sample; *V*_1_ was the volume of the sample consumption of hydrochloric acid standard solution, mL; *V*_2_ was the volume of the hydrochloric acid standard solution consumed by the reagent blank, mL; *C* was the concentration of the hydrochloric acid standard solution, mol/L; and *V* was the volume of the sample, mL.

Purity of Pv was calculated according to formula [33]:

$$P^{*}=N\times 7.69$$

where P* was the purity of the Pv, and *N* was the nitrogen content of the sample.

To determine total phosphorus and inorganic phosphorus in the sample, 1 mL of the Pv sample solution (1 mg/mL) was digested following the method of Zhang et al. [18] and then cooled to room temperature. One milliliter of undigested Pv sample solution (1 mg/mL) was used as the inorganic phosphorus sample. The digested Pv sample and the undigested Pv sample were supplemented to 50 mL by ultrapure water, respectively. One milliliter of the diluted Pv samples solution was mixed with 3 mL of phosphorus reagent (ultrapure water, 6 mol/L sulfuric acid, 2.5% ammonium molybdate solution, 10% ascorbic acid solution, mixed in a ratio of 2:1:1:1), and placed in a 45 °C water bath for 20 min. The absorbance was measured at 660 nm. The phosphorus content was calculated using a standard curve drawn from a standard phosphorus reagent and a phosphorus reagent, while ultrapure water was used instead of the sample as a blank control. The standard phosphorus reagent was prepared by dissolving 0.4389 g of KH_2_PO_4_ in distilled water to 100 mL, and diluted 100 times when used.

Phosphorus content in samples was calculated with the following formula:

$$P=P_{1}-P_{2}$$

where *P* was the phosphorus content in the sample, *P*_1_ was the total phosphorus content in the sample, and *P*_2_ was the content of inorganic phosphorus in the sample.

### 2.4. Determination of Activity

The ELISA is a double antibody sandwich method for determining the concentration of Pv in a sample [34]. Sample extraction of the dilution was carried out as per manufacturer’s instructions of the ELISA kits. The microplates were respectively set with a blank control well (no sample and enzyme standard reagents, the other steps were the same), standard wells and sample wells to be tested. The Pv standard and Pv sample were formulated to the appropriate concentration. Fifty microliters of the corresponding sample was accurately added to the standard wells and the sample wells to be tested, and then blocked for 30 min at 37 °C incubation. The wells were washed with washing liquid five times and wells were blocked with a 50 µL of enzyme standard reagent for 30 min at 37 °C for incubation. After washing with washing liquid five times, 50 μL of developer A and 50 μL of developer B were added to each well and the samples were incubated at 37 °C for 10 min in the dark. Fifty microliters of stop solution was added to each well, and the color developed was read on the microplate reader (Spark 10 M, Tecan, Männedorf, Switzerland) at 405 nm. Activity was calculated with the following formula [35]:

$$A=\frac{C}{C_{0}}\times 100\%$$

where *C* was the concentration of the extracted Pv lyophilized sample measured by the ELISA, (μg/mL); and *C*_0_ was the concentration of the Pv standard measured by the ELISA, (μg/mL).

### 2.5. Sodium Dodecyl Sulfate-Polyacrylamide Gel Electrophoresis (SDS-PAGE) Analysis

The Pv was analyzed by SDS-PAGE (Bio-Rad Co., Ltd., Guangzhou, China) as described by Jiang [36,37]. The resolving gel concentration was 12%, and the concentration of the stacking gel was 5%, and the molecular weight of the pre-stained protein Marker ranged from 14.4~116.0 kDa. The separation process was carried out at a constant voltage of 80 V for stacking gel and 120 V till the dye front reached the bottom of the gel, respectively. After the electrophoresis was completed, the gel was then placed in the fixing solution for 30 min. After being dyed in staining solution 1 at room temperature for 30 min, it was placed in staining solution 2 for another 30 min. Finally, the gel was decolorized in a decolorizing solution, and the decolorizing solution was replaced several times until the background of the gel became shallow [18].

### 2.6. Liquid Chromatography-Nanoelectrospray Ionization Mass Spectrometry (Nano LC-ESI-MS/MS) Analysis

The electrophoresis bands of the purified Pv (Pv lyophilized sample with optimal extraction condition) were cleaned and digested in-gel with sequencing-grade modified trypsin (Promega) in the digestion buffer (ammonium bicarbonate 100 mm, pH 8.5). The peptides from the digestion were extracted out with acetonitrile, and completely dried down in a SpeedVac device [38]. The dried sample was then re-dissolved in solvent (2% acetonitrile, 97.5% ultrapure water and 0.5% formic acid). The polypeptide was identified using the Nano LC-ESI-MS/MS mass spectrometer with an Agilent C18 column (75 μm × 8 cm, 3 μm). The mobile phase A consisted of 97.5% water, 2% acetonitrile and 0.5% formic acid; and mobile phase B was 9.5% water, 90% acetonitrile and 0.5% formic acid. The mobile phase B increased from 2% to 90% in 0~60 min; the loaded sample was 20 min; the column was washed for 20 min; the flow velocity was 0.8 μL/min; and the sample injection volume was 3 μL. The mass spectrometry conditions were a spray voltage of 1.5–1.8 kV, a capillary temperature of 100 °C, a collision energy of 33%, and a microscan mass range of 350–1650 amu [39]. Mass spectrometry data were searched for using the UniProt protein database and analyzed by ProtTech’sProtQuest (Philadelphia, PA, USA) software suite [40].

### 2.7. Fourier-Transform Infrared (FT-IR)

The purified Pv (2 mg) were ground with KBr powder (200 mg) and pressed into a disk of around 1-cm diameter for FT-IR (Bruker ALPHA-T spectrometer, Bruker, Karlsruhe, Germany) measurement between 400 cm^−1^ and 4000 cm^−1^ [41,42]. The infrared spectrum was analyzed by PeakFit v4.12 software to analyze the protein conformation [43].

### 2.8. Iron-Binding Ability of Phosvitin

A mixture of 3.3 mL ultrapure water, 50 μL of 3.0 mm FeCl_3_ (or 3.0 mm FeCl_2_) and 50 μL of 10 mg/mL Pv solution was placed in a tube. The mixture was left at room temperature for 1 min. Four-hundred microliters of 5 mmol/L ferrozine and 200 μL of 1% (*v*/*v*) ascorbic acid were added to the tube. After incubating for 5 min at room temperature, the absorbance was measured at 562 nm, and distilled water was used as a blank [33].

Iron-binding ability was calculated with the following formula:

$$K=\frac{A}{A_{0}}\times 100\%$$

where *K* was the binding ability of Pv with iron ions, *A*_0_ was the absorbance of the blank solution, and *A* was the absorbance of the Pv solution.

### 2.9. Surface Hydrophobicity Measurements

Surface hydrophobicity was determined based on the method of Liu et al. [44]. The Pv standard and purified Pv were prepared into protein solutions with a concentration of 0.1–0.5 mg/mL, respectively. Three milliliters of protein solution with different concentrations was mixed with 12.5 μL of 8 mmol/L 8-anilinonaphtalene-1-sulfonic acid ammonium salt (ANS) solution (prepared with 10 mmol, pH = 7.0 phosphate buffer), and the fluorescence (PerkinElmer LS55, Fremont, CA, USA) intensity of the samples was measured after 15 min. The excitation wavelength was 390 nm, the emission wavelength was 470 nm, and the slit width was 5 nm. The slope obtained from the linear regression of the fluorescence intensity-protein concentration (mg/mL) plots was used as the surface hydrophobicity of Pv.

### 2.10. Statistical Analysis

All the experiments were repeated three times; SPSS version 20.0 was used to analyze the data, which was expressed as the mean ± SD.

## 3. Results and Discussion

### 3.1. Single-Factor Variable Analysis

#### 3.1.1. Effect of Heating Time on Pv Extraction

**Figure 2:**
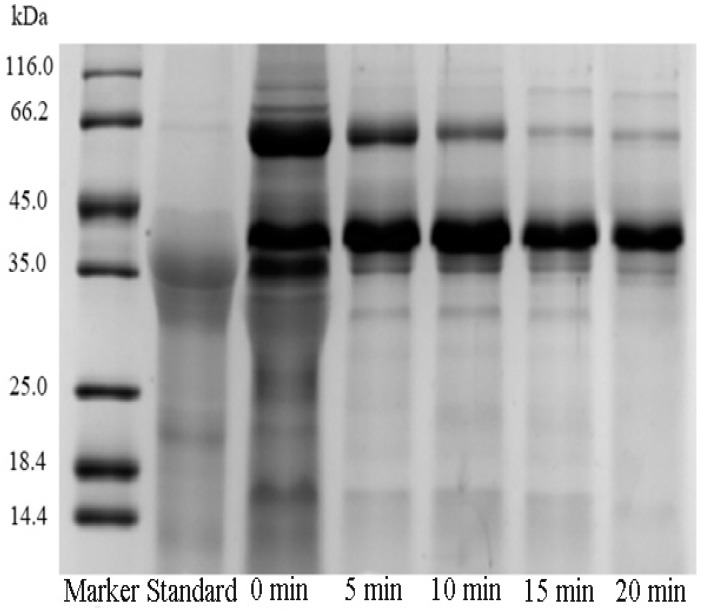
SDS-PAGE of Pv for different heating times (0, 5, 10, 15, and 20 min). Other operating conditions: Heating temperature, 80 °C; ultrasound power, 600 W; and ultrasound time, 10 min.

In the present experiment, the effect of heating time (0, 5, 10, 15, and 20 min) on the extraction of Pv was evaluated. The heating temperature, ultrasound power and ultrasound time amount were set at 80 °C, 600 W and 10 min, respectively. SDS-PAGE of Pv extracted under different heating times is shown in Figure 2, which indicates that the purity of Pv increased as the heating time increased. A major band at approximately 35–45 kDa and two minor bands at 66 kDa were obtained on the lane of the Pv standard. When the heating time was 0 min, six bands could be observed on the electropherogram of the extracted Pv sample. The band at 116, 90, 66, 31, and 15 kDa corresponded to yolk lipoprotein (HDL), and the band at 35–45 kDa corresponded to Pv. When the heating time reached 15 min, the band of HDL was very shallow, while no significant change was obtained when the heating time continued to increase. It might be explained by the fact that low-density lipoprotein and *α*-lipovitellin were denatured during the heating process and removed with precipitation, while *β*-lipovitellin was retained in the solution due to relatively high thermal stability. As shown in Table 1, when the heating time increased from 0 min to 15 min, the purity of Pv increased from 65% to 80% and the activity increased from 66% to 98%, while N/P decreased from 3.8 to 3.1. As the heating time increased continuously, there was no significant change in the purity, N/P and activity of Pv, indicating that it was unnecessary to continue to extend the heating time. Therefore, 15 min was chosen as the optimum heating time.

#### 3.1.2. Effect of Ultrasound Power on Pv Extraction

**Figure 3:**
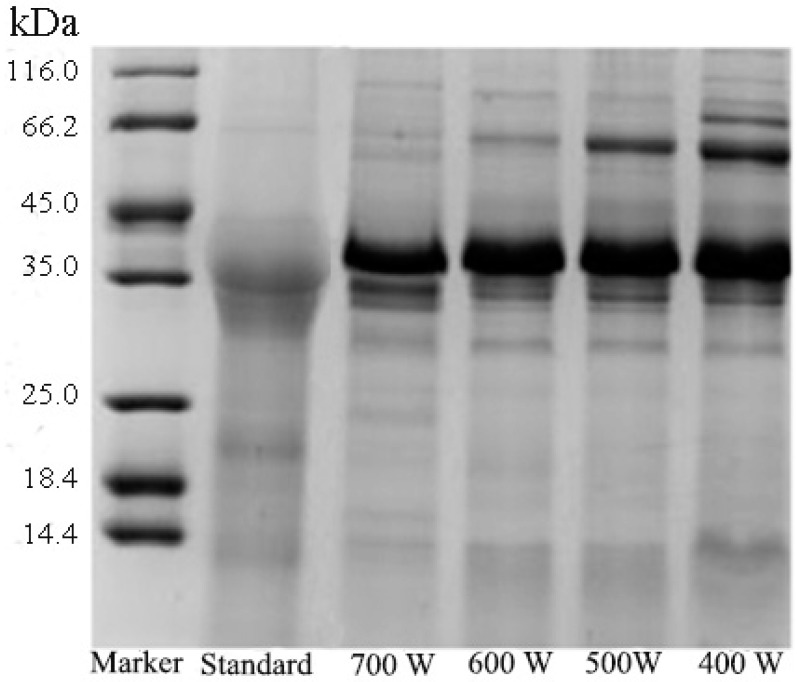
SDS-PAGE of Pv for different ultrasound powers (400, 500, 600, and 700 W). Other operating conditions: Heating temperature, 80 °C; heating time, 15 min; and ultrasound time, 10 min.

The influence of different ultrasound powers (400, 500, 600, and 700 W) on the extraction of Pv was evaluated. Ultrasonic power was related to ultrasonic cavitation, while the higher ultrasound power led to greater intensity of cavitation, causing aggregation and precipitation of HDL [45]. As shown in Figure 3, when the ultrasound power increased from 400 W to 600 W, the band of HDL became lighter, indicating that most of the HDL was removed. When the ultrasound power was above 600 W, the band of Pv was also narrowed. This is probably due to the excessive power caused by the co-precipitation of Pv and HDL. Table 2 showed the effect of ultrasound power on purity, N/P and activity. As the ultrasound power increased from 400 W to 600 W, the purity of Pv increased from 73% to 80% and the activity increased from 72% to 98%, while N/P decreased from 3.6 to 3.1. When the ultrasound power increased above 600W, no significant change was obtained on all the three indexes. In spite of the fact that the band of HDL almost disappeared in SDS-PAGE, there was no significant change in the composition of the extracted Pv sample. Considering the results in Figure 3 and Table 2, 600 W was chosen as the optimized ultrasound power.

#### 3.1.3. Effect of Ultrasound Time on Pv Extraction

**Figure 4:**
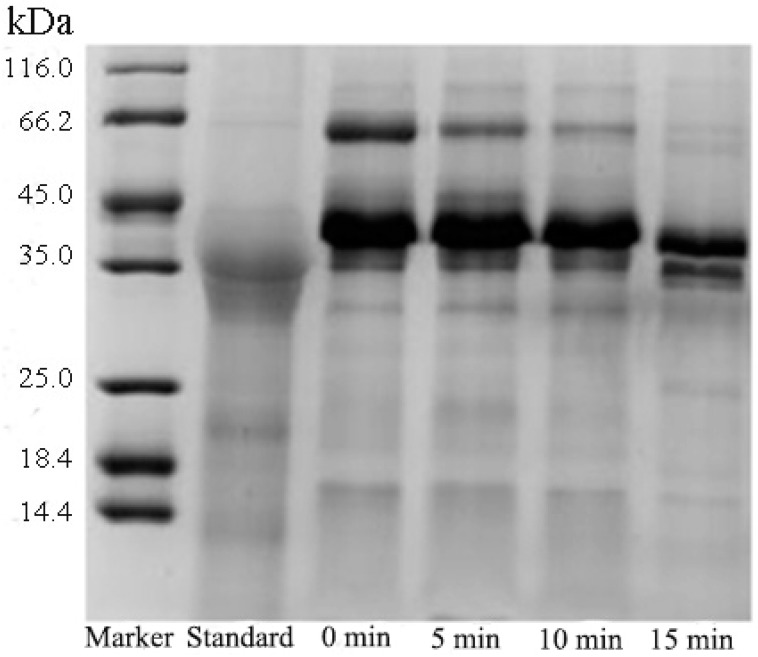
SDS-PAGE of Pv for different ultrasound times (0, 5, 10, and 15 min). Other operating conditions: Heating temperature, 80 °C; heating time, 15 min; and ultrasound power, 600 W.

The effect of ultrasound time on Pv extraction is described in Figure 4 and Table 3. As the ultrasound time increased, the electrophoresis bands became more singular. The amounts of cavitation bubbles produced by the ultrasound increased as the ultrasound time increased, and the instantaneous high temperature caused by the cavitation led to denaturation of HDL. Excessive ultrasound time might cause denaturation of Pv. As a result, the band of Pv was also significantly lightened when the ultrasound time was more than 15 min. Table 3 indicates a similar result, when the ultrasonic time was increased from 0 min to 10 min, the purity and activity were increased, and the N/P was decreased, even if the ultrasonic time continued to increase, the three indicators remained substantially the same. It could be concluded from Figure 4 and Table 3 that moderate ultrasound time facilitated the extraction of Pv.

### 3.2. Identification of Pv by Tandem Mass Spectrometry

Table 4 shows the results of purified Pv and the peptide of Pv determined by Nano LC-ESI-MS/MS. Five proteins were detected, including P02845|VIT2_CHICK (Vitellogenin-2, Gallus gallus), P87498|VIT1_CHICK (Vitellogenin-1, Gallus gallus), P19121, P02752, and P01012. The relative abundance of VIT2 and VIT1 was 52.9% and 37.6% respectively, meaning that Pv was the main protein in the sample. The result is similar to Zhang [46].

**Figure 5:**
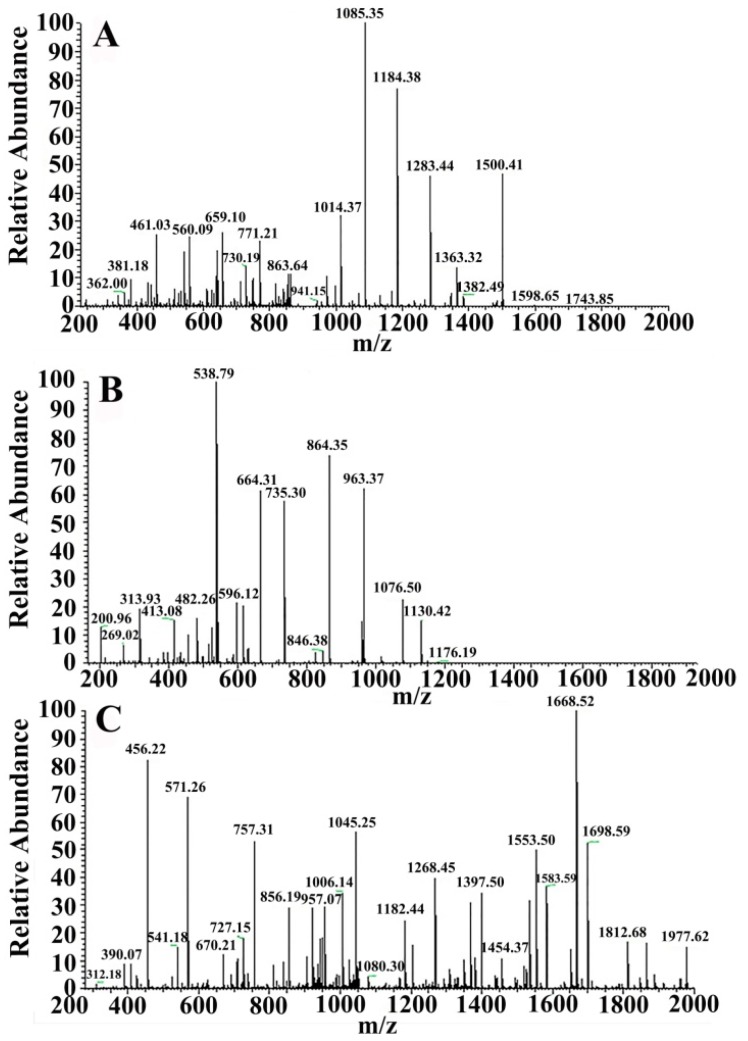
Two-level mass spectrometry of VIT1. (**A**) SCNVVVAQDCTEHPK; (**B**) TVIVEAPIHGLK; (**C**) NPTIDGEESTCYSVDPVLK.

**Figure 6:**
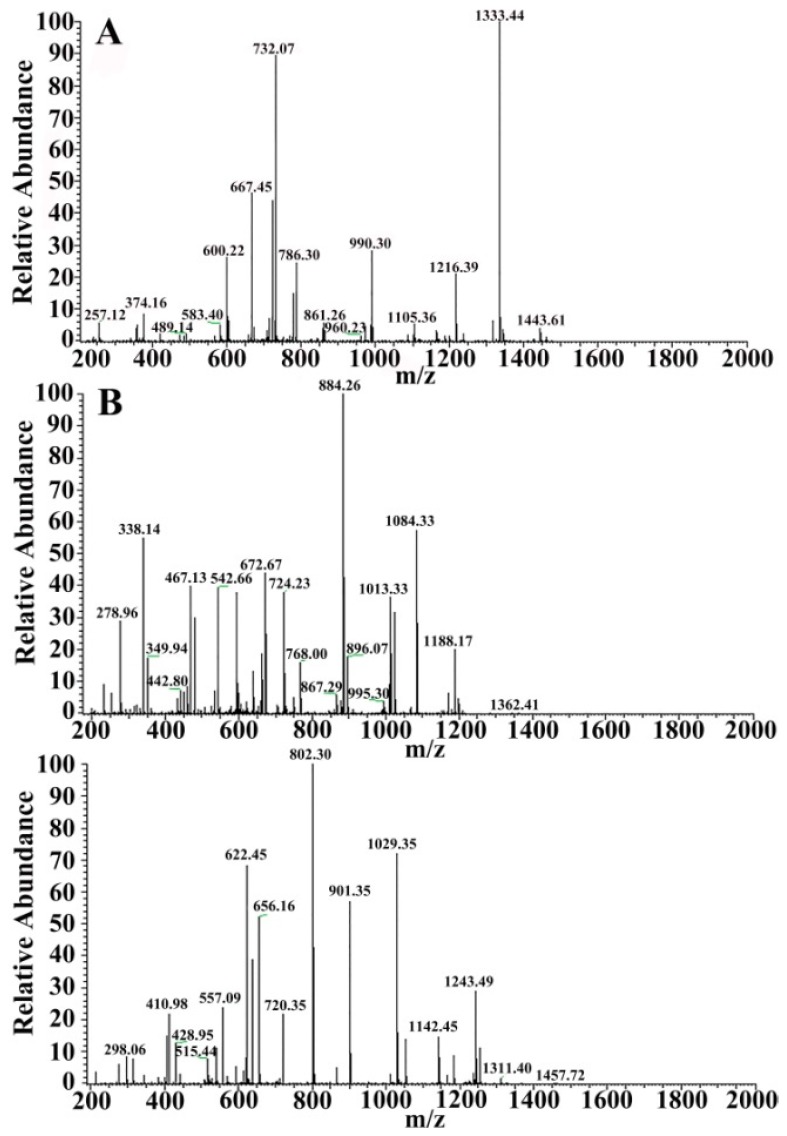
Two-level mass spectrometry of VIT2. (**A**) KKPMDEEENDQVK; (**B**) YDAECEQEYR; (**C**) TITIQVPLWMAGK.

For in solution digestion, a protein solution sample was first reduced by DTT and all Cysteine residues alkylated by iodoacetamide and cleaned by ethanol precipitation. The sample was then digested with sequencing-grade modified trypsin (Promega) in the digestion buffer (ammonium bicarbonate 100 mm, pH 8.5). A dissolved peptide sample was then analyzed by a Nano LC-ESI- MS/MS system. The peptide of VIT1 and VIT2 in the Pv sample identified by Nano LC-ESI-MS/MS was indicated in Table 5 and Table 6. The *m*/*z* was defined as the molecular mass of the peptides measured in mass spectrum. Figure 5 and Figure 6 show the cleavage peptides of VIT1 and VIT2, respectively. In Figure 5: (A) SCNVVVAQDCTEHPK, (B) TVIVEAPIHGLK and (C) NPTIDGEESTCYSVDPVLK, respectively; and Figure 6: (A) KKPMDEEENDQVK, (B) YDAECEQEYR and (C) TITIQVPLWMAGK, respectively.

### 3.3. Infrared Spectra Analysis

**Figure 7:**
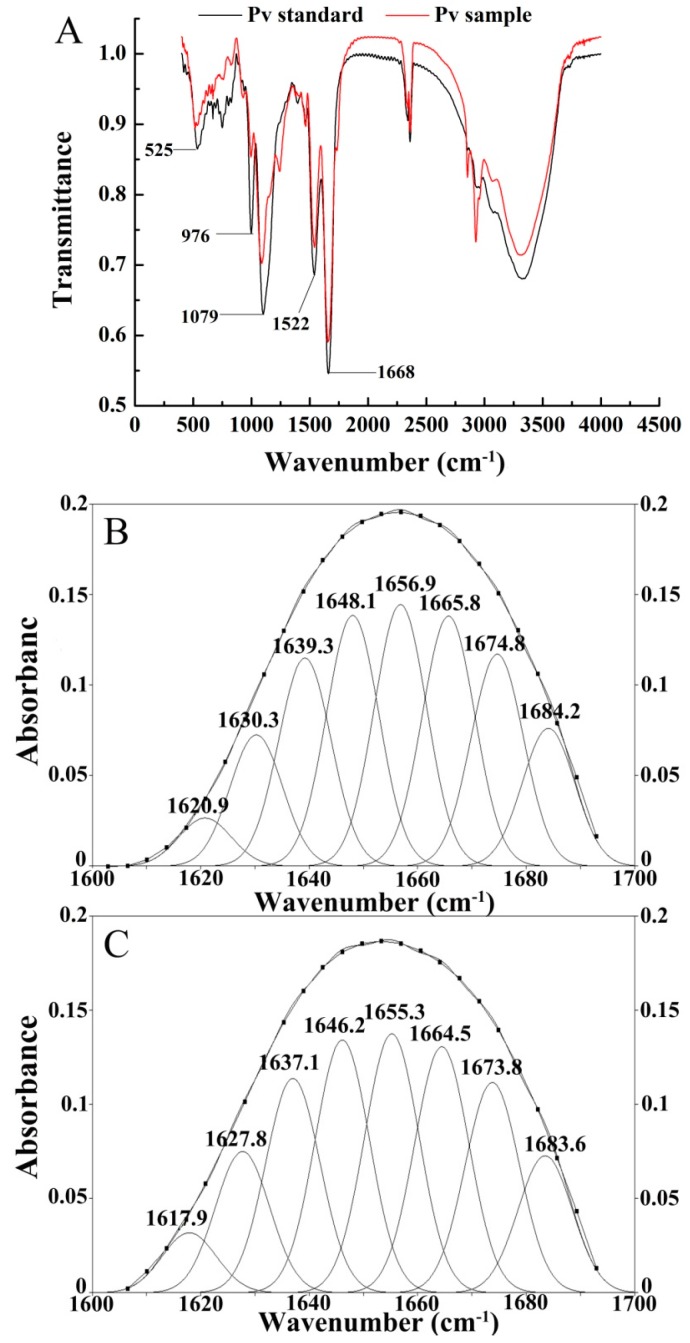
FT-IR of Pv. (**A**) FTIR spectra of Pv standard and purified Pv; (**B**) Fitting FTIR chart for Pv standard; (**C**) Fitting FTIR chart for purified Pv.

As shown in Figure 7A, there was no significant difference in the FT-IR of the Pv standard and purified Pv. The peaks at 525 cm^−1^, 976 cm^−1^ and 1079 cm^−1^ were the bending vibration peak, the symmetric stretching vibration peak and the asymmetric stretching vibration peak of PO_4_^3−^, respectively. The peaks at 1600~1700 cm^−1^ and 1500~1600 cm^−1^ represented the amide I and II bands, respectively, which indicated that Pv is a highly phosphorylated protein. At present, Deconvolve Gaussian IRF combined with second derivative was used to analyze the fit of the amide I absorption band [47]. The bands at 1610–1640 cm^−1^, 1640–1650 cm^−1^, 1650–1660 cm^−1^, and 1660–1700 cm^−1^ represented the *β*-sheet, unordered structure, *α*-helix, and *β*-turn in the amide I band, respectively [48,49]. PeakFit v4.12 software was used to analyze the infrared spectrum amide I absorption bands of Pv standard and purified Pv. The fitted spectrum obtained after baseline correction, Deconvolve Gaussian IRF deconvolution and second derivative fitting are shown in Figure 7B,C. According to the corresponding relationship between each sub-peak and the secondary structure, the integral area was calculated to obtain the relative percentage of the secondary structure, which is shown in Table 7. There was no significant difference in the secondary structure of the Pv standard and the extracted sample. The content of *β*-sheet and *β*-turn of Pv was more than the content of the *α*-helix and unordered structure. It may be due to that the *β*-type conformation is readily acquired by the protein in the absence of iron, which is similar to the study by Taborsky [50] and Renugopalakrishnan et al. [51].

### 3.4. Iron Binding Ability of Pv

The Pv showed a strong iron-binding ability due to its unique phosphorylation structure, and 95% of the iron in the egg yolk was bound with Pv. The binding ability of purified Pv with Fe^2+^ and Fe^3+^ was 27.2% and 28.1%, respectively, and the binding ability of Pv standard with Fe^2+^ and Fe^3+^ was 53.6% and 53.8%, respectively. Choing [52] found that the ability of Pv to bind to iron far exceeded that of transferring; 0.125 mg/mL Pv solution was heated at 110 °C for 20~40 min an did not free iron [53]. However, Hegenauer [54] found that ethylenediaminetetraacetic acid (EDTA) can dissociate bound iron and the reaction process is reversible. As shown in Table 8, the purified Pv condition and Pv standard were not much different in iron-binding ability.

### 3.5. Analysis of Surface Hydrophobicity

The endogenous fluorescence of tyrosine residues and tryptophan residues was quite sensitive to the microenvironmental polarity of protein conformation, so it could be used to reflect the hydrophobic properties of Pv. Table 9 shows that the hydrophobicity of the Pv standard and purified Pv were 488.6 and 493.48, respectively. There was no significant difference in the hydrophobicity of the Pv standard and purified Pv, indicating that the structure of the Pv was not affected during the extraction process.

## 4. Conclusions

In this study, UTAE was used to extract Pv from egg yolk, and the optimum conditions were determined by a single factor. The high purity of Pv (80 ± 2%) showed that UTAE is an effective method for protein extraction. The results of Nano LC-MS/MS mass spectrometry showed that the identified proteins were mainly VIT2 and VIT1, and the relative abundances were 52.9% and 37.6%, respectively. VIT1 and VIT2 are precursors of Pv-chicken VIT. Fluorescence spectroscopy showed that the hydrophobicity of the Pv standard and the sample was almost the same, indicating that the UTAE did not destroy the structure of the Pv during the extraction process. The FT-IR analysis indicated that the UTAE did not change the secondary structure of the Pv in the extraction process.

## Figures and Tables

**Table 1:** Effect of heating time (min) on purity, N/P and activity of Pv.

Heating Time (min)	Purity (%)	N/P	Activity (%)
0	65 ± 1 ^a^	3.8 ± 0.0 ^d^	66 ± 1 ^a^
5	74 ± 1 ^b^	3.6 ± 0.0 ^c^	76 ± 1 ^b^
10	75 ± 1 ^b^	3.4 ± 0.0 ^b^	84 ± 1 ^c^
15	80 ± 2 ^c^	3.1 ± 0.0 ^a^	98 ± 1 ^d^
20	80 ± 1 ^c^	3.2 ± 0.0 ^a^	96 ± 1 ^d^

**Note:** The same letter followed by the same column means that the difference is not significant (*p* > 0.05), and the difference between marked letters indicates that the difference is significant (*p* < 0.05).

**Table 2:** Effect of ultrasound power (W) on purity, N/P and activity of Pv.

Ultrasound Power (W)	Purity (%)	N/P	Activity (%)
400	73 ± 1 ^a^	3.6 ± 0.0 ^c^	72 ± 1 ^a^
500	77 ± 1 ^b^	3.3 ± 0.0 ^b^	88 ± 1 ^b^
600	80 ± 2 ^c^	3.1 ± 0.0 ^a^	98 ± 1 ^c^
700	80 ± 1 ^c^	3.2 ± 0.0 ^a^	97 ± 1 ^c^

**Note:** The same letter followed by the same column means that the difference is not significant (*p* > 0.05), and the difference between marked letters indicates that the difference is significant (*p* < 0.05).

**Table 3:** Effect of ultrasound time (min) on purity, N/P and activity of Pv.

Ultrasound Time (min)	Purity (%)	N/P	Activity (%)
0	72 ± 2 ^a^	3.6 ± 0.0 ^c^	74 ± 1 ^a^
5	77 ± 1 ^b^	3.4 ± 0.0 ^b^	89 ± 1 ^b^
10	80 ± 2 ^c^	3.1 ± 0.0 ^a^	98 ± 1 ^c^
15	80 ± 1 ^c^	3.2 ± 0.0 ^a^	96 ± 1 ^c^

**Note:** The same letter followed by the same column means that the difference is not significant (*p* > 0.05), and the difference between marked letters indicates that the difference is significant (*p* < 0.05).

**Table 4:** The test results of Pv sample by Nano LC-ESI-MS/MS.

Hits	Protein Molecule Weight (Da)	Number of Peptides	Link	Relative Abundance
1	206,730.27	101	P02845	52.9%
2	212,606.75	85	P87498	37.6%
3	71,867.75	5	P19121	1.1%
4	28,276.62	4	P02752	7.8%
5	43,195.66	2	P01012	0.6%

**Table 5:** The peptides of the VIT1 by Nano LC-ESI-MS/MS.

Peptide Number	Amino Acid Sequence	*m*/*z*
5162	KVDHQSLSR	1068.57
6126	EKHNELLMPNHK	1488.75
6134	SCNVVVAQDCTEHPK	1742.77
6147	HNELLMPNHK	1231.60
6315	VGFHCFPK	990.47
6414	ESVLSDSGVSEYEK	1527.68
6454	TVIVEAPIHGLK	1275.75
6462	VTVASWMR	948.48
6469	ESVLSDSGVSEYEKDNIK	1997.93
6504	NVNFDGEILK	1147.59
6548	NPTIDGEESTCYSVDPVLK	2122.97
6694	ATAVSLLEWQR	1272.68
7964	DCTPIEK	861.39

**Table 6:** The peptides of the VIT2 by Nano LC-ESI-MS/MS.

Peptide Number	Amino Acid Sequence	*m*/*z*
5178	SAGEATNLK	889.45
5996	KKPMDEEENDQVK	1588.74
6104	YDAECEQEYR	1361.52
6146	IKTFNEVK	977.55
6165	MPNGYLAK	892.45
6167	TVQLAGVDSK	1016.55
6176	SFVKLEK	849.50
6189	MPNGYLAK	892.45
6247	CYSTEPVLR	1123.53
6264	IGSHEIDMHPVNGQVK	1759.86
6387	SAGEATNLKAINIK	1428.79
6584	TTPVTVGFHCLPADSANSLTDK	2330.12
6606	SAGEATNLKAINIKIGSHEIDMHPVNGQVK	3170.65
6742	NAVSFGHSWILEEAPCR	1971.93
7027	YDAECEQEYR	1361.52
7251	CYSTEPVLR	1123.53
7466	TVQLAGVDSK	1016.54

**Table 7:** Content of the secondary structure for Pv standard and purified Pv.

Source	*α*-Helix (%)	*β*-Sheet (%)	*β*-Turn (%)	Unordered (%)
Pv standard	17.4 ± 0.14 ^a^	26.4 ± 0.12 ^a^	39.5 ± 0.11 ^a^	16.7 ± 0.14 ^a^
Purified Pv	17.1 ± 0.11 ^a^	27.3 ± 0.13 ^a^	39.0 ± 0.14 ^a^	16.6 ± 0.15 ^a^

**Note:** The same letter followed by the same column means that the difference is not significant (*p* > 0.05), and the difference between marked letters indicates that the difference is significant (*p* < 0.05).

**Table 8:** The Pv standard and purified Pv with Iron-binding ability.

Source	Bind with Fe^2+^	Bind with Fe^3+^
Pv standard	28.1 ± 0.72 ^a^	53.8 ± 1.11 ^a^
purified Pv	27.2 ± 0.65 ^a^	53.6 ± 1.1 ^a^

**Note:** The same letter followed by the same column means that the difference is not significant (*p* > 0.05), and the difference between marked letters indicates that the difference is significant (*p* < 0.05).

**Table 9:** Hydrophobicity of Pv standard and purified Pv.

Source	Surface Hydrophobicity
Pv standard	488.6 ± 2.14 ^a^
purified Pv	493.48 ± 2.31 ^a^

**Note:** The same letter followed by the same column means that the difference is not significant (*p* > 0.05), and the difference between marked letters indicates that the difference is significant (*p* < 0.05).
